# GLP-1-Based Therapies for Cardiovascular Risk Reduction in Kazakhstan: Evidence, Affordability, Equity and Implementation Considerations

**DOI:** 10.3390/jcm15208190

**Published:** 2026-10-21

**Authors:** Kulzhan Berikkhanova, Isah Inuwa, Yessenkhan Sultan, Nurzhan Berikkhanov, Saule Rakhimova, Ainur Akilzhanova

**Affiliations:** 1 National Laboratory Astana, Nazarbayev University, Astana 010000, Kazakhstan; 2 University Medical Center, Nazarbayev University, Astana 010000, Kazakhstan; 3 Professor G.V. Tsoi Scientific and Educational Center of Surgery, Astana Medical University, Astana 010000, Kazakhstan; 4 Medical Center “Sultan-Medicus”, Almaty 050000, Kazakhstan; 5 City Polyclinic No. 2, Taraz 080000, Kazakhstan

**Keywords:** Central Asia, glucagon-like peptide-1 receptor agonists, cardiovascular disease, Kazakhstan, metabolic correction, MACE, cardiometabolic risk

## Abstract

Cardiovascular disease (CVD) remains the leading cause of mortality worldwide and is the principal cause of death in low- and middle-income countries. Although cardiovascular outcome trials have established the benefits of Glucagon-like peptide-1 (GLP-1) receptor agonists (GLP-1RAs) in reducing major adverse cardiovascular events, the translation of this evidence into routine clinical practice in Central Asia remains limited and underexplored. Specifically, in Kazakhstan, despite measurable improvements in cardiac infrastructure over the past decade, critical gaps persist in early diagnosis, cardiometabolic risk stratification, and the integration of newer pharmacological strategies into routine practice. In this review, we aim to evaluate the potential role of GLP-1-based therapies within the current cardiovascular prevention landscape in Kazakhstan. It also synthesizes current mechanistic evidence and findings from major clinical trials supporting the use of GLP-1RAs as cardiometabolic agents with potential cardiovascular benefits. Furthermore, we examine the practical factors that may influence their adoption within Kazakhstan’s health system, including accessibility, affordability, clinician preparedness, prescribing capacity, regulatory considerations, and policy integration. By integrating evidence from cardiovascular outcome trials, clinical studies, and health-system perspectives, this review seeks to provide a balanced assessment of the potential contribution of GLP-1-based therapies to cardiovascular risk reduction in Kazakhstan while identifying priorities for future research, implementation strategies, and health policy development.

## 1. Introduction

Cardiovascular diseases (CVDs) remain the leading cause of mortality in Kazakhstan and represent a major health burden across Central Asia, where obesity, diabetes, hypertension, and other cardiometabolic disorders continue to challenge cardiovascular prevention and long-term disease management [1,2]. Although improvements in cardiovascular care infrastructure have occurred in recent years, important gaps remain in early detection, risk stratification, equitable access to advanced therapies, and integration of newer pharmacological approaches into routine clinical practice [3,4]. These challenges make Kazakhstan an important setting for examining how emerging cardiometabolic therapies can be translated from clinical trial evidence into real-world healthcare systems. Globally, CVDs remain the leading cause of death, accounting for approximately 19.8 million deaths in 2022, with a disproportionate burden occurring in low- and middle-income countries where prevention, diagnosis, and treatment capacity remain constrained [5,6]. In Kazakhstan, historical cardiovascular mortality rates have been reported at nearly twice the European average, reflecting the persistent impact of cardiometabolic risk factors and healthcare-system challenges [6].

Cardiovascular diseases (CVDs) remain the leading cause of death worldwide, claiming nearly 20 million lives each year. These disorders, ranging from heart attacks and strokes to heart failure and peripheral artery disease, continue to pose a challenge to public health, driving both mortality and long-term disability across populations [7,8]. In 2022, an estimated 19.8 million people died from CVDs, representing nearly one-third of all global mortality, with the majority of deaths occurring in low- and middle-income countries where early diagnosis and management often lag behind global standards [5]. The burden is particularly pronounced in Kazakhstan, where historical cardiovascular mortality rates have been reported at nearly twice the European average [6]. This disproportionate burden, combined with evolving cardiometabolic risk profiles and healthcare-system challenges, makes Kazakhstan a relevant setting for evaluating how emerging cardiovascular therapies can be translated into clinical practice.

Glucagon-like peptide-1 receptor agonists (GLP-1RAs), class B G-protein-coupled receptor agonists, have progressed beyond their original role as glucose-lowering therapies for type 2 diabetes to become important cardiometabolic agents with demonstrated cardiovascular, weight-management, and kidney-related benefits [9,10,11,12]. Although cardiovascular outcome trials have established the benefits of Glucagon-like peptide-1 (GLP-1) receptor agonists (GLP-1RAs) in reducing major adverse cardiovascular events, their implementations in Central Asia remain limited and underexplored.

Despite these important advances, the available trial evidence addresses a narrower question than its potential clinical impact in Kazakhstan may suggest. Cardiovascular outcome trials, including LEADER [13], SUSTAIN-6 [14], REWIND [15], and SELECT [16,17] establish efficacy under controlled conditions characterized by randomized patient selection, structured follow-up, and reliable treatment delivery. However, these trials do not determine which populations should be prioritized when healthcare resources are constrained; whether observed benefits are transferable to Central Asian populations with different metabolic profiles and comorbidity patterns; whether treatment is affordable and sustainable within national healthcare budgets; how long therapy should be continued to maintain clinical benefit; how patients outside major urban centres can access the monitoring required for safe use; or whether investment in GLP-1 receptor agonists may divert resources from other high-value cardiovascular interventions. Translating trial efficacy into real-world health-system benefit therefore represents a distinct challenge from demonstrating efficacy itself.

Kazakhstan represents a transitional health system between high-income and low- and middle-income settings, combining a relatively developed healthcare infrastructure with persistent cardiometabolic risk and regional inequities. This position makes Kazakhstan a scientifically informative setting in which to examine the translation of emerging cardiometabolic therapies into routine cardiovascular care. The country faces a substantial and evolving cardiometabolic and cardiovascular disease burden [6,18], with marked urban–rural differences in mortality patterns and risk-factor distribution [19]. However, healthcare capacity remains unevenly distributed, with specialist services and physician resources concentrated in urban centres, limiting access to advanced chronic disease management in rural and remote communities [20,21]. The national digital health infrastructure, including the Unified National Electronic Healthcare System, is expanding and may support improved longitudinal monitoring and integrated care delivery [22]. Nevertheless, real-world evidence on the implementation and outcomes of GLP-1 receptor agonists in Kazakhstan remains limited. These features, including post-Soviet health-system structures, healthcare inequalities, and evolving chronic disease management capacity, are shared across many Central Asian countries, making Kazakhstan a relevant case study for exploring how emerging cardiometabolic therapies can be translated into equitable population-level practice.

Existing studies of GLP-1RAs have largely addressed mechanisms of action, the comparative profiles of individual drugs, the results of major cardiovascular outcome trials, or their role in diabetes and obesity management. What is largely missing is an integrated assessment that connects the cardiovascular evidence to population phenotype, health-system capacity, affordability, equity, and implementation feasibility in a Central Asian setting specifically. This review is intended to address that gap.

Accordingly, this review addresses four key questions. First, what cardiovascular benefits of GLP-1-based therapies are supported by current clinical evidence, and which patient populations derive the greatest benefit? Second, how applicable is the existing evidence base to Kazakhstan and Central Asian populations with different epidemiological and healthcare contexts? Third, what factors related to affordability, accessibility, equity, and healthcare capacity may influence adoption of these therapies? Fourth, what research priorities and implementation approaches are justified given the current evidence gaps and regional healthcare realities? In addressing these questions, this review does not treat GLP-1RAs as a complete solution to Kazakhstan’s cardiovascular burden—barriers to access, gaps in long-term safety evidence, and the absence of locally generated trial data all warrant caution—but argues that the available evidence justifies their serious consideration within future cardiovascular prevention and treatment strategy.

## 2. Materials and Methods

This narrative review followed a structured evidence synthesis approach. The literature was identified through PubMed, Scopus, Web of Science, and Google Scholar from inception to July 2026. Search terms included combinations of “GLP-1 receptor agonists,” “cardiovascular outcome trials,” “cardiovascular disease,” “cardiometabolic risk,” “Kazakhstan,” “Central Asia,” “implementation,” “affordability,” “equity,” and the generic and trade names of individual GLP-1-based agents.

## 3. Epidemiology and Magnitude of the Cardiovascular Burden

Kazakhstan carries one of the heaviest CVD burdens in Central Asia. According to the World Health Organization and national surveillance data, CVD mortality rates in Kazakhstan have historically exceeded those of most European countries by a factor of approximately two, and the country has ranked among the highest in the Central Asian region for CVD-related mortality [18].

Kassymkhan et al. (2026), in their study of cardiovascular disease burden in Central Asia, reported that Kazakhstan experienced a steady decline in overall CVD mortality between 2021 and 2023, followed by a slight increase in 2024 [19]. Urban populations demonstrated higher mortality rates than rural ones. Hence, this finding may reflect the greater metabolic impact of urban dietary and lifestyle patterns, as well as potential ascertainment biases in rural reporting. Ischaemic heart disease, including acute myocardial infarction (AMI), accounts for the single largest share of CVD mortality, contributing substantially to disability-adjusted life years (DALYs) in the 45–74 age group [23,24].

Additionally, data from the Unified National Electronic Healthcare System (UNEHS) in Kazakhstan, covering the period 2014–2019 and subsequently updated, reveal a rising prevalence of heart failure as both a primary diagnosis and a secondary contributor to hospitalisation. This trend mirrors patterns seen in high-income countries but occurs in a health system that faces more constrained resources for chronic disease management [22].

## 4. Risk Factors Driving Cardiovascular Disease in Kazakhstan

Cardiovascular disease in Kazakhstan is driven by a complex interaction of metabolic, behavioural, environmental, and healthcare-system factors [1,25]. Although improvements in cardiovascular care have contributed to declining mortality trends, ischaemic heart disease and stroke remain major causes of morbidity and mortality. The most important modifiable risk factors include hypertension, dyslipidaemia, excess body weight, diabetes, unhealthy dietary patterns, tobacco exposure, and physical inactivity [25,26]. These factors often cluster together, creating a cardiometabolic profile associated with accelerated atherosclerosis, myocardial infarction, stroke, heart failure, and premature mortality. Regional inequalities, urbanisation, and unequal access to preventive healthcare further influence the distribution and management of cardiovascular risk across the country [1,27,28].

Obesity, diabetes, and metabolic dysfunction are particularly relevant to the potential application of GLP-1-based therapies in Kazakhstan because these conditions represent major drivers of cardiovascular risk and are key therapeutic targets of GLP-1 receptor agonists. National data indicate substantial burdens of excess weight and dyslipidaemia, with overweight and obesity contributing to insulin resistance, hypertension, inflammation, and increased cardiovascular risk [26,29,30,31]. Similarly, diabetes prevalence has increased substantially over recent decades, exposing a growing population to diabetes-associated vascular complications. Since type 2 diabetes commonly coexists with obesity and hypertension, cardiovascular prevention increasingly requires glucose-lowering strategies selected according to overall cardiovascular and renal risk rather than glycaemic control alone [32,33,34,35,36,37].

Behavioural and environmental factors, including unhealthy dietary patterns, high sodium intake, tobacco use, physical inactivity, and air pollution, further contribute to Kazakhstan’s cardiovascular burden [1,34,38,39,40,41,42,43,44,45]. These factors highlight that GLP-1-based therapies should not be considered as replacements for established prevention strategies, including lifestyle modification, blood-pressure control, lipid management, and smoking cessation. Instead, they may represent an additional component of integrated cardiometabolic risk reduction, particularly among patients with obesity, type 2 diabetes, established cardiovascular disease, or high cardiovascular risk.

## 5. Physiological and Pharmacological Background of GLP-1

Glucagon-like peptide-1 (GLP-1) is an incretin hormone secreted primarily by enteroendocrine L-cells of the distal small intestine and colon in response to nutrient ingestion. It plays an important role in glucose homeostasis by potentiating glucose-stimulated insulin secretion from pancreatic β-cells and suppressing inappropriate glucagon release from pancreatic α-cells [46,47]. These complementary actions reduce postprandial hyperglycaemia while limiting the risk of excessive insulin release during periods of low blood glucose. GLP-1 also delays gastric emptying and promotes satiety through peripheral and central nervous system pathways, contributing to reduced food intake and weight loss [48].

The therapeutic potential of endogenous GLP-1 is limited due to its rapid enzymatic degradation, primarily by dipeptidyl peptidase-4 (DPP-4). GLP-1 receptor agonists (GLP-1RAs) were developed to reproduce the physiological effects of GLP-1 while maintaining a longer duration of action [49]. These agents may be classified according to their pharmacokinetic profiles. Short-acting agents, such as exenatide and lixisenatide, have a pronounced effect on postprandial glucose control [50]. Longer-acting agents, including liraglutide, dulaglutide, and semaglutide, provide more sustained receptor activation and stronger effects on fasting glucose control [51]. More recently, GLP-1-based therapies have expanded to include multi-receptor agonists such as tirzepatide, which activates both the glucose-dependent insulinotropic polypeptide (GIP) receptor and the GLP-1 receptor [52].

For the purposes of this review, selective GLP-1 receptor agonists (GLP-1RAs) are distinguished from multi-receptor incretin therapies. Selective GLP-1RAs, including liraglutide, semaglutide, dulaglutide, exenatide, lixisenatide, albiglutide, and efpeglenatide, primarily activate the GLP-1 receptor. Tirzepatide is classified separately as a dual glucose-dependent insulinotropic polypeptide (GIP)/GLP-1 receptor agonist because its pharmacological activity extends beyond selective GLP-1 receptor activation. The broader term “GLP-1-based therapies” is used in this review only when the discussion encompasses both selective GLP-1RAs and multi-receptor incretin therapies. Cardiovascular efficacy, safety, and implementation considerations are therefore interpreted according to the specific pharmacological class and available evidence for individual agents.

## 6. Molecular Mechanism of Action of GLP-1 Receptor Agonists

**Figure 1:**
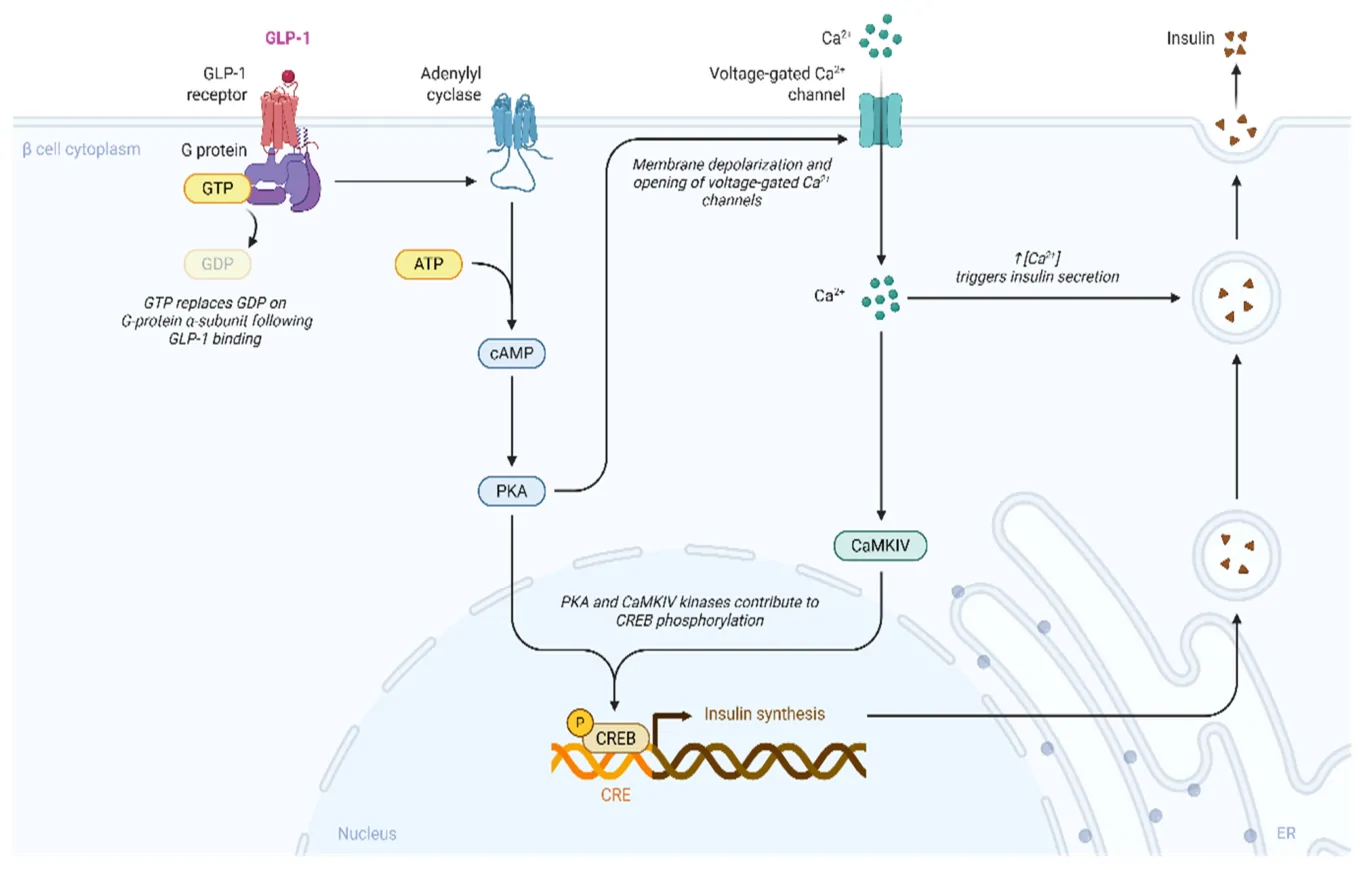
Mechanism of action of GLP-1. Binding of GLP-1 or a GLP-1 receptor agonist to the G-protein-coupled GLP-1 receptor activates adenylyl cyclase, increases intracellular cAMP, and stimulates PKA-dependent and calcium-dependent signalling. Calcium influx through voltage-gated channels promotes insulin-granule exocytosis, while CREB phosphorylation supports insulin synthesis and CaMKIV contributes to the regulation of insulin gene transcription. Abbreviation: ATP, adenosine triphosphate; cAMP, cyclic adenosine monophosphate; CREB, cAMP-responsive element-binding protein; CaMKIV, calcium/calmodulin-dependent protein kinase IV; GDP, guanosine diphosphate; GLP-1, glucagon-like peptide-1; PKA, protein kinase A. Created in BioRender. Inuwa, I. (2026) https://BioRender.com/ixsfet1 (accessed on 22 August 2026).

GLP-1RAs exert their effects by binding to and activating the GLP-1R, a class B G-protein-coupled receptor expressed on pancreatic β-cells and various extra-pancreatic tissues [48]. As illustrated in Figure 1, GLP-1 receptor activation stimulates the Gαs protein, which activates adenylyl cyclase and increases intracellular cyclic adenosine monophosphate (cAMP) [53]. The rise in cAMP activates protein kinase A (PKA) and other downstream signalling mediators, including exchange protein directly activated by cAMP 2 (Epac2) [54]. These pathways amplify glucose-dependent insulin secretion rather than initiating insulin release independently of glucose concentration [55].

Activation of cAMP-dependent signalling promotes membrane depolarisation and facilitates the opening of voltage-gated calcium channels [56]. The resulting influx of calcium ions increases exocytosis of insulin-containing secretory granules. Calcium-dependent signalling also contributes to cAMP-responsive element-binding protein (CREB) phosphorylation, which supports insulin gene transcription and biosynthesis [57]. Consequently, GLP-1 receptor activation produces both an immediate increase in glucose-dependent insulin secretion and a longer-term enhancement of β-cell secretory capacity [58].

Beyond pancreatic β-cell signalling, GLP-1RAs suppress glucagon secretion in a glucose-dependent manner, reducing hepatic glucose output during hyperglycaemia [59]. They also delay gastric emptying, moderating postprandial glucose entry, and act at hypothalamic centres that reduce appetite and energy intake [60]. Together, these mechanisms improve glycaemic control and contribute to clinically significant weight reduction.

The cardiovascular benefits of GLP-1 receptor agonists (GLP-1RAs) are supported by established clinical effects and a range of proposed metabolic, haemodynamic, renal, inflammatory, and direct cardiovascular mechanisms. However, the relative contribution of these mechanisms and the cellular localization of canonical GLP-1 receptors within human cardiovascular tissues remain incompletely resolved. Human tissue studies have detected full-length GLP1R transcripts in all four cardiac chambers but have not definitively localized translated GLP-1R protein to specific ventricular cell types. The same studies did not detect GLP1R transcripts in human coronary endothelial or vascular smooth muscle cells, whereas other immunohistochemical studies have reported GLP-1R localization in cardiac myocytes and the sinoatrial node. These differences indicate that receptor localization may depend on tissue, species, experimental method, and the sensitivity and specificity of receptor detection techniques [61].

Human experimental studies nevertheless provide evidence for cardiovascular actions beyond glucose lowering. In patients with coronary artery disease, GLP-1 administration reduced coronary microvascular resistance and increased coronary flow, despite an absence of detectable GLP-1R in vascular tissue, suggesting that coronary effects may involve myocardial-vascular crosstalk rather than direct vascular receptor activation [62]. More recently, ex vivo experiments using isolated human ventricular cardiomyocytes reported that semaglutide reduced late sodium current and diastolic sarcoplasmic reticulum calcium leak while improving calcium transients and contractility. These effects were attenuated by GLP-1R antagonism, providing experimental evidence for a direct myocardial action, although such findings should not be interpreted as resolving the broader controversy surrounding GLP-1R localization in human ventricular tissue [63].

Additional endothelial, vascular smooth muscle, and immune-cell mechanisms have mainly been demonstrated in experimental systems. In cultured human aortic endothelial cells, liraglutide reduced inflammatory responses and altered calcium-dependent AMPK and eNOS signalling [64]. In cultured vascular smooth muscle cells and experimental vascular injury models, exendin-4 reduced proliferation and neointimal formation, with evidence implicating GLP-1R-dependent signalling [65]. Similarly, experimental studies in macrophages and atherosclerosis models have reported reduced inflammatory signalling and monocyte/macrophage accumulation following GLP-1R agonist treatment [66]. These findings provide plausible mechanisms for cardiovascular protection, but they remain predominantly preclinical or translational and should not be presented as established mechanisms of cardiovascular benefit in humans.

Accordingly, the established cardiovascular effects of GLP-1RAs should be distinguished from proposed direct cellular mechanisms. Current clinical evidence supports cardiovascular risk reduction with selected GLP-1-based therapies, whereas the precise contribution of direct cardiomyocyte, endothelial, vascular smooth muscle, and immune-cell GLP-1R signalling remains an active area of investigation.

In experimental models, GLP-1R agonists such as exendin-4 have reduced vascular smooth muscle cell proliferation, senescence, and neointimal formation through pathways involving cAMP/PKA and related signalling mechanisms. However, the presence and functional significance of canonical GLP-1R in human vascular smooth muscle remain incompletely established [65]. Experimental studies have also reported anti-inflammatory effects of GLP-1R agonists in macrophages, including reductions in inflammatory cytokine expression, NF-κB signalling, monocyte adhesion, and foam-cell formation. These findings suggest a possible contribution of immune-cell modulation to vascular protection, although the extent to which this represents a direct GLP-1R-dependent mechanism in human atherosclerotic tissue remains uncertain [66].

Experimental studies suggest that GLP-1RAs may influence cardiomyocyte calcium handling, mitochondrial function, and oxidative stress. However, the extent to which these effects reflect direct canonical GLP-1R signalling in human ventricular cardiomyocytes remains under investigation. Recent ex vivo evidence with semaglutide provides support for direct effects on calcium and sodium handling in human ventricular cardiomyocytes [63].

## 7. Biological Rationale for Metabolic Correction Through GLP-1 Receptor Agonists

The cardiovascular relevance of glucagon-like peptide-1 receptor agonists (GLP-1 RAs) extends well beyond glucose lowering and appears to involve interacting metabolic, haemodynamic, renal, inflammatory, and potentially direct vascular pathways. First, the substantial weight reduction produced by some GLP-1 RAs addresses obesity, an important upstream driver of insulin resistance, hypertension, systemic inflammation, obstructive sleep apnoea, and obesity-related heart failure. In the STEP 5 trial, once-weekly semaglutide 2.4 mg produced substantial weight loss that was sustained over 104 weeks [67]. More importantly, the SELECT trial demonstrated that semaglutide reduced the incidence of cardiovascular death, nonfatal myocardial infarction, or nonfatal stroke by 20% among adults with established cardiovascular disease and overweight or obesity but without diabetes [17]. These findings support the treatment of excess adiposity as an important component of cardiovascular risk reduction, although the extent to which the observed cardiovascular benefit was mediated by weight loss rather than other drug effects remains uncertain.

Second, GLP-1 RAs improve glycaemic regulation by enhancing glucose-dependent insulin secretion, suppressing inappropriate glucagon release, and reducing postprandial glucose exposure. Improved glycaemic control can reduce glucotoxic stress and contribute to the prevention of diabetes-related microvascular complications. However, the cardiovascular benefits observed in outcome trials should not be attributed solely to reductions in glycated haemoglobin, because cardiovascular protection has also been demonstrated in people without diabetes, as shown in SELECT [17].

Third, GLP-1 RA treatment is commonly accompanied by modest reductions in systolic blood pressure and improvements in several cardiometabolic indicators. In the STEP-HFpEF trial, semaglutide treatment yielded greater reductions in body weight, systolic blood pressure, and C-reactive protein than placebo and improved symptoms, physical limitations, and exercise capacity among patients with obesity-related heart failure with preserved ejection fraction [68]. These haemodynamic and anti-inflammatory changes may contribute to cardiovascular benefit, although they are unlikely to explain the entire treatment effect.

Fourth, kidney protection may provide an additional pathway through which GLP-1 RAs reduce cardiovascular risk. Albuminuria and declining kidney function are strongly associated with adverse cardiovascular outcomes. In the FLOW trial, semaglutide reduced the risk of major kidney disease events and death from cardiovascular causes among patients with type 2 diabetes and chronic kidney disease [69]. The treatment also slowed the decline in estimated glomerular filtration rate, providing evidence of clinically meaningful cardiorenal protection rather than an effect limited to changes in albuminuria alone [69].

In addition to their metabolic and renal effects, emerging experimental and translational evidence suggests that GLP-1 RAs may exert direct vascular effects, including improvement in endothelial function, reduction in oxidative stress, suppression of vascular inflammation, and inhibition of atherosclerotic plaque development [70,71]. Human experimental research has reported direct vasodilatory and endothelial effects, while animal models have shown reductions in atherosclerotic lesion formation partly independent of changes in body weight or cholesterol levels [72,73]. Nevertheless, evidence for these direct mechanisms remains less definitive than the evidence from major cardiovascular and kidney outcome trials. Their relative contribution, compared with indirect improvements in body weight, blood pressure, glycaemic regulation, lipid metabolism, inflammation, and kidney function, remains incompletely resolved.

Cardiometabolic risk reduction through GLP-1 RAs should therefore be understood as a multidimensional process rather than simply an extension of glucose lowering. These agents should not be regarded as substitutes for established cardiovascular prevention and treatment, including healthy dietary patterns, physical activity, smoking cessation, blood-pressure control, lipid-lowering treatment, and other evidence-based therapies. Instead, current evidence indicates that appropriately selected GLP-1 RAs can serve as important components of integrated cardiometabolic and cardiorenal care, particularly for patients with type 2 diabetes, obesity, established atherosclerotic cardiovascular disease, or chronic kidney disease.

## 8. Pivotal Cardiovascular Outcome Trials

Eleven pivotal cardiovascular outcome trials relevant to GLP-1-based therapies were identified and are summarized in Table 1. These trials include studies of selective GLP-1 receptor agonists as well as the more recent SURPASS-CVOT evaluating the dual GIP/GLP-1 receptor agonist tirzepatide. The cardiovascular findings are therefore interpreted according to the pharmacological class of each agent rather than assumed to represent a uniform class effect.

## 9. Evidence from Meta-Analyses

Sattar et al. (2021) [81] pooled data from eight large cardiovascular outcome trials involving 60,080 participants with type 2 diabetes. GLP-1 receptor agonists reduced three-point major adverse cardiovascular events (MACE) by 14% (HR 0.86, 95% CI 0.80–0.93), all-cause mortality by 12% (HR 0.88, 95% CI 0.82–0.94), and hospital admission for heart failure by 11% (HR 0.89, 95% CI 0.82–0.98). They also reduced a broad composite kidney outcome by 21% (HR 0.79, 95% CI 0.73–0.87). However, this kidney composite included new-onset macroalbuminuria as well as deterioration in kidney function, kidney replacement therapy, and kidney-related death. It should therefore not be interpreted as a 21% reduction in kidney failure alone.

Badve et al. (2025) [82] subsequently analysed 11 randomised trials involving 85,373 participants, including data from the FLOW and SELECT trials. Among the 67,769 participants with type 2 diabetes, GLP-1 receptor agonists reduced the primary composite kidney outcome by 18% (HR 0.82, 95% CI 0.73–0.93), kidney failure by 16% (HR 0.84, 95% CI 0.72–0.99), MACE by 13% (HR 0.87, 95% CI 0.81–0.93), and all-cause mortality by 12% (HR 0.88, 95% CI 0.83–0.93). When participants without diabetes from SELECT were included, the corresponding reductions were 19% for the composite kidney outcome, 16% for kidney failure, 14% for MACE, and 13% for all-cause mortality. Although the overall incidence of serious adverse events did not differ significantly from placebo, discontinuation because of adverse events was more frequent with GLP-1 receptor agonists (RR 1.51, 95% CI 1.18–1.94).

More recently, Galli et al. (2025) [83] evaluated 21 randomised trials involving 99,599 participants across populations with diabetes, obesity, chronic kidney disease, heart failure, or elevated cardiovascular risk. The analysis included eight agents, including tirzepatide, which is a dual glucose-dependent insulinotropic polypeptide and GLP-1 receptor agonist rather than a selective GLP-1 receptor agonist. Treatment was associated with reductions of 12% in all-cause mortality, 13% in cardiovascular mortality, 13% in MACE, 15% in myocardial infarction, and 15% in heart-failure hospitalisation. Gastrointestinal disorders increased by 63%, while gallbladder-related disorders increased by 26%. The reported 9% reduction in acute kidney failure was only borderline and did not reach conventional statistical significance (IRR 0.91, 95% CI 0.83–1.00; *p* = 0.053); it should therefore be described as a numerical rather than a definitive reduction.

Collectively, these meta-analyses provide consistent evidence that selective GLP-1 receptor agonists reduce major cardiovascular events, mortality, heart-failure hospitalisation, and selected kidney outcomes in high-risk populations. More recent analyses incorporating multi-receptor incretin therapies, including tirzepatide, provide additional evidence of cardiovascular and cardiometabolic benefit, but these findings should be interpreted separately because the pharmacological profiles of multi-receptor incretin therapies differ from those of selective GLP-1RAs. The available evidence therefore supports cardiovascular benefit across specific incretin-based agents but should not be interpreted as demonstrating a uniform effect across all GLP-1-based therapies.

## 10. Safety and Tolerability

The use of GLP-1 RAs requires individualised patient assessment, appropriate dose escalation, patient education, and continued clinical follow-up. Gastrointestinal symptoms—including nausea, vomiting, diarrhoea, constipation, abdominal pain, and dyspepsia—are the most frequently reported adverse effects. They are generally mild to moderate, occur most often during treatment initiation or dose escalation, and frequently diminish with continued treatment. Nevertheless, persistent or severe gastrointestinal symptoms can impair treatment adherence and are an important cause of dose interruption or permanent discontinuation [84,85].

GLP-1 RA treatment is also associated with a modestly increased risk of gallbladder and biliary disorders. A meta-analysis of 76 randomised trials involving 103,371 participants found a 37% relative increase in the combined risk of gallbladder or biliary disease, although the absolute increase was relatively small, amounting to approximately 27 additional events per 10,000 treated people annually. The risk was greater in trials using higher doses, longer treatment durations, and obesity-treatment regimens [86]. A more recent meta-analysis that included both selective GLP-1 receptor agonists and multi-receptor incretin therapies similarly reported increased gastrointestinal and gallbladder-related disorder [83]. Because the analysis included agents with different receptor profiles, these findings should not be interpreted as demonstrating an identical safety profile across all incretin-based therapies.

Clinical monitoring should therefore focus on the severity and persistence of symptoms rather than routine investigation of every patient. Persistent vomiting or diarrhea can cause dehydration, volume depletion, and deterioration in kidney function, particularly during treatment initiation and dose escalation. Patients should be advised to maintain adequate fluid intake and promptly report prolonged gastrointestinal symptoms. Renal function should be assessed when substantial fluid loss, reduced oral intake, or symptoms of acute kidney injury occur. Severe or persistent abdominal pain should also prompt evaluation for pancreatitis, while right-upper-quadrant pain, fever, jaundice, or other suggestive symptoms should lead to investigation for gallbladder disease [84].

Additional precautions depend on the individual drug, treatment indication, dose, and accompanying medications. The risk of hypoglycaemia is generally low when GLP-1 RAs are used alone but increases when they are combined with insulin or insulin-secretagogue therapies such as sulfonylureas. Dose adjustment and glucose monitoring may therefore be required in such patients. Particular caution may also be appropriate in patients with severe gastrointestinal motility disorders, previous pancreatic or gallbladder disease, advanced frailty, or diabetic retinopathy, in accordance with the prescribing information for the selected agent and relevant national clinical guidance [84].

These safety considerations become important when GLP-1-based therapies are introduced at population scale. A national programme should not concentrate exclusively on medicine procurement and affordability. It should also establish transparent eligibility and exclusion criteria, standardised dose-escalation procedures, prescriber training, patient counselling materials, scheduled clinical follow-up, and clear pathways for treatment interruption, specialist assessment, or referral when complications occur. Integration with the national pharmacovigilance system is essential for the systematic detection, assessment, and reporting of suspected adverse drug reactions and emerging safety signals [87].

National implementation should also include safeguards against inappropriate prescribing, counterfeit products, and unsupervised use. Safety communication must remain responsive to evolving evidence because some risks may be specific to particular agents rather than representing a class effect. For example, WHO currently regards non-arteritic anterior ischaemic optic neuropathy as a very rare safety signal driven mainly by semaglutide data; evidence is not yet sufficient to establish it as a class-wide effect. Patients using semaglutide should nevertheless be advised to report sudden visual changes promptly [88].

Overall, GLP-1 RAs have a favourable benefit–risk profile for appropriately selected patients, but safe implementation depends on careful patient selection, gradual dose escalation, early management of adverse effects, ongoing pharmacovigilance, and accessible referral services. These arrangements are especially important in Kazakhstan if selective GLP-1RAs and multi-receptor incretin therapies are to be incorporated into broader cardiometabolic and cardiovascular risk-reduction programmes, with safety monitoring adapted to the specific agent and treatment indication.

## 11. Clinical Assessment and Risk-Stratification Relevance

Glucagon-like peptide-1 receptor agonists (GLP-1 RAs) are therapeutic rather than diagnostic agents. Nevertheless, incorporating them into cardiovascular care requires a structured assessment of cardiometabolic and cardiorenal risk so that treatment is directed towards patients with appropriate clinical indications based on available evidence and guideline recommendations rather than functioning as a universal treatment approach. In people with established cardiovascular disease or a high cardiovascular risk profile, assessment should include body weight and body mass index, with waist circumference measured where clinically feasible; blood pressure; fasting plasma glucose or glycated haemoglobin; a complete lipid profile; and kidney function assessed using serum creatinine and estimated glomerular filtration rate. Urinary albumin-to-creatinine ratio should also be measured, particularly in patients with diabetes, hypertension, or suspected chronic kidney disease, because albuminuria provides important prognostic information even when estimated glomerular filtration rate remains relatively preserved [89,90].

Risk assessment should extend beyond laboratory measurements. A comprehensive cardiovascular evaluation should also consider clinical characteristics, treatment feasibility, and factors that may influence access to care. Clinicians should document smoking status, dietary practices, physical activity levels, alcohol use, current medications, and relevant socioeconomic or treatment-access barriers. Clinical history and examination should also evaluate symptoms and signs suggestive of heart failure, including exertional breathlessness, fatigue, peripheral oedema, orthopnoea, and reduced exercise tolerance. Previous myocardial infarction, angina, coronary revascularisation, stroke, transient ischaemic attack, peripheral arterial disease, heart failure, and chronic kidney disease should be recorded because these conditions substantially influence cardiovascular risk classification and the selection of glucose-lowering and weight-management therapies. Current guidance supports the use of a GLP-1 RA with demonstrated cardiovascular benefit in appropriately selected people with type 2 diabetes and established atherosclerotic cardiovascular disease, multiple cardiovascular risk factors, or chronic kidney disease, irrespective of whether additional glucose lowering is required [89,90].

Beyond conventional cardiometabolic parameters, emerging evidence suggests that systemic nutritional status may provide additional prognostic information in cardiovascular risk assessment. Nutritional indices that integrate parameters such as serum albumin and lymphocyte count have been associated with adverse cardiovascular outcomes in selected clinical populations, highlighting the potential value of considering nutritional status alongside traditional risk factors. Recent evidence from patients undergoing coronary artery bypass grafting demonstrated that the Prognostic Nutritional Index provided additional prognostic information for in-hospital mortality beyond established surgical risk models, although further validation across broader cardiovascular populations is required [91].

In Kazakhstan, this structured assessment is particularly relevant because cardiometabolic risk factors may be under-recognised or treated separately rather than incorporated into an integrated cardiovascular risk profile. However, Kazakhstan-specific evidence regarding GLP-1 RA prescribing patterns, reimbursement coverage, treatment affordability, and cost-effectiveness remains limited. Therefore, implementation decisions should be interpreted cautiously and informed by both international evidence and local healthcare-system considerations. GLP-1 RA eligibility should therefore not be determined by body weight or glycated haemoglobin alone. Treatment decisions should consider the combined presence of obesity, diabetes, hypertension, dyslipidaemia, kidney dysfunction, albuminuria, established atherosclerotic cardiovascular disease, and heart-failure risk. This approach represents a risk-stratification framework rather than a prescriptive treatment algorithm and requires further validation using real-world Kazakhstan-specific data. When appropriately validated, it may improve patient selection, identify untreated risk factors, establish appropriate baseline measurements, and provide objective indicators for monitoring therapeutic response.

## 12. Conceptual Framework for Translating GLP-1-Based and Multi-Receptor Incretin Therapies into Cardiovascular Care

Translating evidence from GLP-1-based therapies into cardiovascular practice requires a structured implementation approach that distinguishes selective GLP-1 receptor agonists from multi-receptor incretin therapies. In low- and middle-income countries, including Kazakhstan and other Central Asian settings, implementation should consider the specific cardiovascular evidence, indications, safety profile, affordability, and evidence base of each therapeutic class. Selective GLP-1RAs with demonstrated cardiovascular benefit should be considered according to established clinical indications, while newer multi-receptor incretin therapies such as tirzepatide should be evaluated according to their emerging cardiovascular evidence and distinct pharmacological profile. Equally important are strategies to address healthcare access disparities, particularly in rural and underserved regions, through strengthened referral pathways, telemedicine support, and specialist collaboration. The proposed framework (Table 2) is conceptual and is intended to identify potential implementation priorities rather than provide a validated treatment pathway.

### Kazakhstan-Specific Implementation Context

The feasibility of translating GLP-1-based cardiovascular evidence into practice depends on the country’s regulatory, financing, prescribing, and medicine-access environment. Kazakhstan’s National Drug Formulary includes several selective GLP-1 receptor agonists, including exenatide, liraglutide, dulaglutide, and semaglutide [92]. However, inclusion in the national formulary does not necessarily indicate universal outpatient access or reimbursement for every clinical indication. The current outpatient medicine-coverage arrangements should therefore be distinguished from formulary status when assessing access.

The national burden of diabetes provides an important context for implementation. Ministry of Health data indicate that 535,033 patients with diabetes were registered in Kazakhstan in 2025, while annual financing for diabetes treatment was approximately 45.5 billion tenge [93]. Figures demonstrate substantial existing health-system investment but do not provide a GLP-1-specific budget-impact estimate. Similarly, national data describing GLP-1RA prescribing volumes, patient-level out-of-pocket expenditure, rural availability, and regional variation in access remain limited.

Regulatory access to newer incretin therapies is also evolving. As of 4 August 2026, the Ministry of Health reported that tirzepatide (Mounjaro) and semaglutide (Wegovy) were undergoing registration procedures [93], their future role in routine cardiovascular and metabolic care should not be assumed to reflect established national practice. These gaps highlight the need for Kazakhstan-specific studies of medicine availability, reimbursement, prescribing patterns, affordability, rural access, and budget impact before large-scale implementation decisions are made.

Given the limited Kazakhstan-specific evidence on GLP-1 prescribing patterns, rural availability, patient out-of-pocket expenditure, agent-specific reimbursement, and budget impact, this framework should be regarded as a hypothesis-generating implementation model rather than a validated national pathway. Future research should evaluate these parameters using real-world Kazakhstan data before national scale-up.

## 13. Discussion

This review demonstrates that GLP-1 receptor agonists (GLP-1RAs) represent an important advance in cardiometabolic medicine, with cardiovascular benefits demonstrated across several clinically relevant populations. However, the strength of evidence varies between individual agents and clinical contexts. Cardiovascular outcome trials have established significant reductions in major adverse cardiovascular events (MACE) with liraglutide, subcutaneous semaglutide, dulaglutide, albiglutide, and efpeglenatide, whereas other agents have primarily demonstrated cardiovascular safety without superiority over placebo [13,14,15,77]. Accordingly, cardiovascular benefits demonstrated by individual agents should not automatically be generalized across all GLP-1-based therapies.

The therapeutic relevance of GLP-1-based therapies has expanded beyond glucose lowering. The SELECT trial demonstrated that semaglutide reduced cardiovascular events in adults with overweight or obesity and established cardiovascular disease without diabetes, extending the potential role of GLP-1-based therapies into obesity-related cardiovascular risk management [16]. Similarly, the SOUL trial strengthened the evidence base by demonstrating cardiovascular benefit with oral semaglutide among high-risk adults with type 2 diabetes [79]. Together, these findings suggest that the clinical importance of GLP-1-based therapies extends beyond glycaemic control and includes integrated management of obesity, diabetes, kidney disease, and cardiovascular risk. However, the extent to which these benefits arise from weight reduction, improved metabolic control, renal protection, direct vascular effects, or combined mechanisms remains incompletely understood.

Given the increasing cardiometabolic burden in Kazakhstan, therapies capable of addressing multiple cardiovascular risk pathways simultaneously may have clinical relevance. Although GLP-1-based therapies have demonstrated cardiovascular benefits internationally, their population-level impact in Kazakhstan will depend on whether healthcare systems can overcome barriers related to cost, availability, clinician familiarity, and long-term treatment access. Therefore, the central challenge is not simply whether GLP-1-based therapies are effective, but whether their demonstrated benefits can be translated into equitable and sustainable cardiovascular care within the Kazakhstan healthcare context.

Addressing this translational challenge requires consideration of how healthcare-system capacity, resource limitations, and local disease patterns influence the implementation of GLP-1-based therapies in Kazakhstan. The country continues to experience a substantial burden of cardiovascular disease, driven by a combination of hypertension, obesity, diabetes, smoking, dietary risk factors, and other cardiometabolic determinants. These risks often cluster within individuals, creating a population profile in which therapies capable of targeting multiple cardiometabolic pathways may provide additional value. However, a high cardiovascular disease burden alone does not justify widespread adoption of GLP-1-based therapies. Their implementation should occur within a structured cardiovascular prevention framework that complements, rather than replaces, established interventions, including blood-pressure control, lipid management, smoking cessation, dietary improvement, physical activity promotion, and early detection of cardiometabolic risk.

The appropriate role of GLP-1-based therapies in Kazakhstan should therefore be based on phenotype-guided treatment rather than broad population prescribing. Priority should be considered for patients with the highest expected absolute benefit, including: (1) individuals with type 2 diabetes and established atherosclerotic cardiovascular disease [89]; (2) individuals with type 2 diabetes and chronic kidney disease [69]; (3) adults with obesity and established cardiovascular disease who meet evidence-based treatment criteria; (4) selected patients with obesity-related heart failure with preserved ejection fraction under appropriate clinical supervision; and (5) individuals with persistent high cardiovascular risk despite optimized standard therapy and lifestyle intervention [68]. Such an approach maximizes clinical benefit while reducing unnecessary expenditure and inappropriate exposure among lower-risk populations.

This risk-based strategy aligns with contemporary international recommendations. The 2023 European Society of Cardiology guidelines, corrected in 2024, recommend the use of GLP-1 receptor agonists with demonstrated cardiovascular benefit in individuals with type 2 diabetes and established atherosclerotic cardiovascular disease, independent of baseline glycated haemoglobin levels or the need for additional glucose reduction [89]. This represents an important shift from glucose-centred treatment decisions toward cardiovascular-risk-based therapeutic selection. However, translating these recommendations into Kazakhstan requires consideration of local disease patterns, healthcare capacity, reimbursement structures, and patient access.

A major unresolved challenge is economic feasibility. Cardiovascular outcome trials demonstrate efficacy under conditions of reliable drug availability, structured follow-up, and high treatment adherence, which may not reflect routine healthcare environments where medication affordability influences initiation and long-term persistence [94]. Although GLP-1-based therapies may provide substantial benefits for appropriately selected patients, their cost may limit population-level accessibility, particularly in healthcare systems with competing priorities [95]. Future implementation strategies in Kazakhstan should therefore incorporate formal health-economic evaluations to determine cost-effectiveness, reimbursement approaches, and optimal patient prioritization. Investment in GLP-1-based therapies should complement rather than displace other high-value cardiovascular interventions with established population-level benefits.

Equitable access represents another critical consideration. Healthcare resources and specialist services remain unevenly distributed across Kazakhstan, with greater concentration in urban centres and continuing challenges in rural and remote regions [96]. Because GLP-1-based therapies require appropriate patient selection, dose escalation, monitoring, and management of adverse effects, unequal healthcare capacity may unintentionally widen existing disparities if implementation is limited to populations with better access to specialist care. Strategies to reduce this risk should include strengthening primary-care prescribing capacity, developing referral pathways, expanding telemedicine support, and integrating digital health systems to facilitate longitudinal monitoring. Without such measures, the introduction of advanced cardiometabolic therapies may benefit mainly urban populations while leaving vulnerable groups behind.

The biological mechanisms underlying cardiovascular protection provide additional insights but also highlight remaining uncertainties. Experimental and translational studies suggest that GLP-1RAs may improve endothelial function, reduce oxidative stress, suppress vascular inflammation, enhance cardiac metabolism, and influence atherosclerotic processes [70,97]. However, these mechanisms remain less definitively established than clinical outcome evidence from randomized trials. The relative contribution of direct vascular effects compared with indirect effects mediated through weight reduction, improved insulin sensitivity, blood-pressure reduction, lipid changes, and kidney protection remains an important area for future investigation.

For Kazakhstan, locally generated evidence remains a critical priority. Although cardiovascular outcome trials provide robust evidence of efficacy, uncertainties remain regarding treatment persistence, real-world safety, affordability, and healthcare-system feasibility within Central Asian populations. Future implementation research should therefore focus on establishing real-world evidence platforms, evaluating treatment patterns and outcomes, and assessing models of care that integrate primary-care capacity, digital health support, and equitable access pathways.

The proposed framework highlights that successful translation of GLP-1-based therapies requires alignment between clinical evidence and health-system readiness, while recognizing that implementation considerations may differ between selective GLP-1RAs and multi-receptor incretin therapies. Decisions regarding treatment selection, affordability, safety monitoring, and reimbursement should therefore be informed by the evidence and pharmacological characteristics of each agent rather than by assuming a uniform class of effect.

## 14. Conclusions

GLP-1 receptor agonists represent an important advancement in cardiometabolic medicine, with robust evidence demonstrating cardiovascular benefits across multiple high-risk populations, including individuals with type 2 diabetes, established cardiovascular disease, obesity, and chronic kidney disease. However, translating these benefits from clinical trials into population-level cardiovascular risk reduction requires more than evidence of biological efficacy. It requires alignment between patient phenotype, healthcare capacity, affordability, equity, safety monitoring, and long-term treatment sustainability.

Kazakhstan provides a relevant case study for examining this translational challenge. Although the country faces a substantial cardiometabolic disease burden and has growing healthcare infrastructure, no cardiovascular outcome trial has specifically evaluated the effectiveness, safety, or long-term impact of GLP-1 receptor agonists in Kazakhstan or broader Central Asian populations. Consequently, uncertainties remain regarding treatment persistence, real-world effectiveness, tolerability, cost-effectiveness, and the extent to which findings from international trials can be generalized to local populations with distinct demographic, metabolic, and healthcare-system characteristics.

Future implementation of GLP-1-based therapies in Kazakhstan should therefore be accompanied by locally generated evidence. Establishing national cardiometabolic registries, strengthening pharmacovigilance systems, and developing real-world evidence platforms will be essential to monitor cardiovascular outcomes, treatment adherence, safety signals, healthcare access, and economic impact. These efforts should be integrated with strategies that address urban–rural disparities, strengthen primary-care capacity, and ensure that treatment prioritization is guided by cardiovascular risk rather than socioeconomic advantage.

Importantly, the term GLP-1-based therapies encompasses agents with different pharmacological profiles. Selective GLP-1RAs should be distinguished from multi-receptor incretin therapies such as tirzepatide when interpreting cardiovascular efficacy, safety, and implementation evidence. This distinction is particularly important for Kazakhstan, where decisions regarding treatment prioritization, reimbursement, monitoring, and health-system integration should be based on agent-specific evidence.

Ultimately, the successful adoption of GLP-1-based therapies in Kazakhstan and Central Asia will depend not only on access to effective medicines but also on the ability to build evidence-driven, equitable, and sustainable models of care. The priority should therefore shift from simply introducing GLP-1 therapies to developing the health-system foundations required to deliver their cardiovascular benefits effectively and fairly at population scale.

## Figures and Tables

**Table 1:** Pivotal cardiovascular outcome trials.

Trial	Intervention/Pharmacological Class	Population	N	Comparator	Follow-Up	Primary Cardiovascular Endpoint	HR (95% CI)	Interpretation	Ref.
ELIXA	Lixisenatide, selective GLP-1RA	T2DM with recent ACS	6068	Placebo	25 months	CV death, MI, stroke, or hospitalization for unstable angina	1.02 (0.89–1.17)	Non-inferior, not superior	[74]
LEADER	Liraglutide, selective GLP-1RA	T2DM and high CV risk	9340	Placebo	3.8 years	3-point MACE	0.87 (0.78–0.97)	Non-inferior and superior	[13]
SUSTAIN-6	Semaglutide SC, selective GLP-1RA	T2DM and high CV risk	3297	Placebo	2.1 years	3-point MACE	0.74 (0.58–0.95)	Non-inferior and superior	[14]
EXSCEL	Exenatide ER, selective GLP-1RA	T2DM with/without prior CVD	14,752	Placebo	3.2 years	3-point MACE	0.91 (0.83–1.00)	Non-inferior, not superior	[75]
Harmony Outcomes	Albiglutide, selective GLP-1RA	T2DM with established CVD	9463	Placebo	1.6 years	3-point MACE	0.78 (0.68–0.90)	Non-inferior and superior	[76]
REWIND	Dulaglutide, selective GLP-1RA	T2DM with prior CVD or CV risk factors	9901	Placebo	5.4 years	3-point MACE	0.88 (0.79–0.99)	Non-inferior and superior	[15]
PIONEER 6	Oral semaglutide, selective GLP-1RA	T2DM and high CV risk	3183	Placebo	15.9 months	3-point MACE	0.79 (0.57–1.11)	Non-inferior, not superior	[77]
AMPLITUDE-O	Efpeglenatide, selective GLP-1RA	T2DM with CVD or CKD	4076	Placebo	1.81 years	3-point MACE	0.73 (0.58–0.92)	Non-inferior and superior	[78]
SELECT	Semaglutide 2.4 mg, selective GLP-1RA	Overweight/obesity with established CVD, without diabetes	17,604	Placebo	39.8 months	3-point MACE	0.80 (0.72–0.90)	Superior	[17]
SOUL	Oral semaglutide, selective GLP-1RA	T2DM with ASCVD and/or CKD	9650	Placebo	49.5 months	3-point MACE	0.86 (0.77–0.96)	Superior	[79]
SURPASS-CVOT	Tirzepatide, dual GIP/GLP-1RA	T2DM with established ASCVD	13,299	Dulaglutide	4.0 years	3-point MACE	0.92 (0.83–1.01)	Non-inferior, not superior	[80]

**Table 2:** Conceptual Framework for Translating GLP-1-Based and Multi-Receptor Incretin Therapies into Cardiovascular Care.

Priority Area	Evidence-Informed Considerations	Potential Benefit
Early cardiometabolic screening	Integrate blood pressure, BMI, waist circumference, HbA1c, lipid profile, kidney function, albuminuria, smoking history, and dietary assessment into routine cardiovascular care.	Earlier identification of high-risk patients
Phenotype-guided treatment	Prioritize GLP-1 RAs with demonstrated cardiovascular benefit for patients with T2DM and atherosclerotic CVD, obesity and established CVD, CKD, or selected HFpEF phenotypes.	More efficient targeting of treatment
Standard-care integration	Combine GLP-1-based therapies with statins, antihypertensive therapy, smoking cessation, dietary modification, physical activity, and other evidence-based treatments.	Avoidance of fragmented care
Rural and regional access	Develop referral pathways, telemedicine support, and specialist consultation models for underserved regions.	Reduction of geographic inequalities
Safety monitoring	Establish adverse-event documentation, dose-titration protocols, discontinuation criteria, and referral pathways.	Improved tolerability and patient safety
Local outcomes registry	Monitor cardiovascular events, body weight, glycemic control, kidney outcomes, treatment persistence, safety, and cost-effectiveness.	Real-world evidence for national decision-making
Research and precision medicine	Evaluate genomic profiles, molecular biomarkers, responder characteristics, and locally relevant risk models.	Improved patient selection
Health-economic evaluation	Assess affordability, reimbursement pathways, and long-term cost-effectiveness.	Sustainable implementation

## Data Availability

No new data were created or analyzed in this study. Data sharing is not applicable to this article.

## Abbreviations

The following abbreviations are used in this manuscript:

AMI	Acute Myocardial Infarction
ATP	Adenosine Triphosphate
BMI	Body Mass Index
cAMP	Cyclic Adenosine Monophosphate
CC BY	Creative Commons Attribution License
CKD	Chronic Kidney Disease
CVDs	Cardiovascular Diseases
DALYs	Disability-Adjusted Life Years
DPP-4	Dipeptidyl Peptidase-4
ECG	Electrocardiogram
eGFR	Estimated Glomerular Filtration Rate
eNOS	Endothelial Nitric Oxide Synthase
Epac2	Exchange Protein Directly Activated by cAMP 2
FDA	Food and Drug Administration
GIP	Glucose-Dependent Insulinotropic Polypeptide
GLP-1	Glucagon-Like Peptide-1
GLP-1R	Glucagon-Like Peptide-1 Receptor
GLP-1RA/GLP-1RAs	Glucagon-Like Peptide-1 Receptor Agonist(s)
HbA1c	Glycated Haemoglobin
HFpEF	Heart Failure with Preserved Ejection Fraction
HR	Hazard Ratio
IL-1β	Interleukin-1 Beta
IL-18	Interleukin-18
KATP	ATP-Sensitive Potassium Channel
LMICs	Low- and Middle-Income Countries
MACE	Major Adverse Cardiovascular Events
NO	Nitric Oxide
NLRP3	NOD-Like Receptor Family Pyrin Domain-Containing 3 Inflammasome
PKA	Protein Kinase A
SGLT2	Sodium-Glucose Cotransporter-2
SOD	Superoxide Dismutase
T2DM	Type 2 Diabetes Mellitus
UN-EHS	Unified National Electronic Healthcare System
VSMCs	Vascular Smooth Muscle Cells
WHO	World Health Organization
WHO STEPS	World Health Organization STEPwise Approach to Noncommunicable Disease Risk Factor Surveillance
RCTs	Randomized Controlled Trials
LEADER	Liraglutide Effect and Action in Diabetes: Evaluation of Cardiovascular Outcome Results
SUSTAIN-6	Semaglutide Unabated Sustainability in Treatment of Type 2 Diabetes
REWIND	Researching Cardiovascular Events with a Weekly INcretin in Diabetes
SELECT	Semaglutide Effects on Heart Disease and Stroke in Patients with Overweight or Obesity
SOUL	Semaglutide Cardiovascular Outcomes Trial
FLOW	Evaluate Renal Function with Semaglutide Once Weekly
STEP-HFpEF	Semaglutide Treatment Effect in People with Obesity and HFpEF
STEP-5	Semaglutide Treatment Effect in People with Obesity
ELIXA	Evaluation of Lixisenatide in Acute Coronary Syndrome
EXSCEL	Exenatide Study of Cardiovascular Event Lowering Trial
AMPLITUDE-O	Effect of Efpeglenatide on Cardiovascular Outcomes
SURPASS-CVOT	Study of Tirzepatide Compared with Dulaglutide on Cardiovascular Events
